# New web-based applications for mechanistic case diagramming

**DOI:** 10.3402/meo.v19.24708

**Published:** 2014-07-23

**Authors:** Fred R. Dee, Thomas H. Haugen, Clarence D. Kreiter

**Affiliations:** 1 Department of Pathology, Carver College of Medicine, University of Iowa, Iowa City, IA, USA; 2 Veterans Affairs Medical Center, Iowa City, IA, USA; 3 Department of Family Medicine, Carver College of Medicine, University of Iowa, Iowa City, IA, USA

**Keywords:** Mechanisms of Disease, Pathogenesis, Pathophysiology, Case Based Learning, Clinical-Pathologic Correlation

## Abstract

The goal of mechanistic case diagraming (MCD) is to provide students with more in-depth understanding of cause and effect relationships and basic mechanistic pathways in medicine. This will enable them to better explain how observed clinical findings develop from preceding pathogenic and pathophysiological events. The pedagogic function of MCD is in relating risk factors, disease entities and morphology, signs and symptoms, and test and procedure findings in a specific case scenario with etiologic pathogenic and pathophysiological sequences within a flow diagram. In this paper, we describe the addition of automation and predetermined lists to further develop the original concept of MCD as described by Engelberg in 1992 and Guerrero in 2001. We demonstrate that with these modifications, MCD is effective and efficient in small group case-based teaching for second-year medical students (ratings of ~3.4 on a 4.0 scale). There was also a significant correlation with other measures of competency, with a ‘true’ score correlation of 0.54. A traditional calculation of reliability showed promising results (α =0.47) within a low stakes, ungraded environment. Further, we have demonstrated MCD's potential for use in independent learning and TBL. Future studies are needed to evaluate MCD's potential for use in medium stakes assessment or self-paced independent learning and assessment. MCD may be especially relevant in returning students to the application of basic medical science mechanisms in the clinical years.

## Introduction

In 1992, Engelberg described ‘etiologic cause and effect diagrams’ for use in lectures to discuss complex medical cases (1). Later in 2001, Guerrero described a similar pedagogy which he labeled ‘mechanistic case diagramming’, and observed that the diagrams serve ‘to foster integration across disciplines and depth in learning the scientific basis for the patient's symptoms, clinical signs and laboratory findings’. These diagrams were used in the re-formulative phase of problem-based learning (PBL) (2). Similarly, Azer in 2005 described the use of ‘diagrammatic mechanisms’ in PBL as a tool to ‘enhance (student's) ability to explain the scientific basis of the symptoms and clinical signs of the patient enlisted in the case’ (3).

**Fig. 1:**
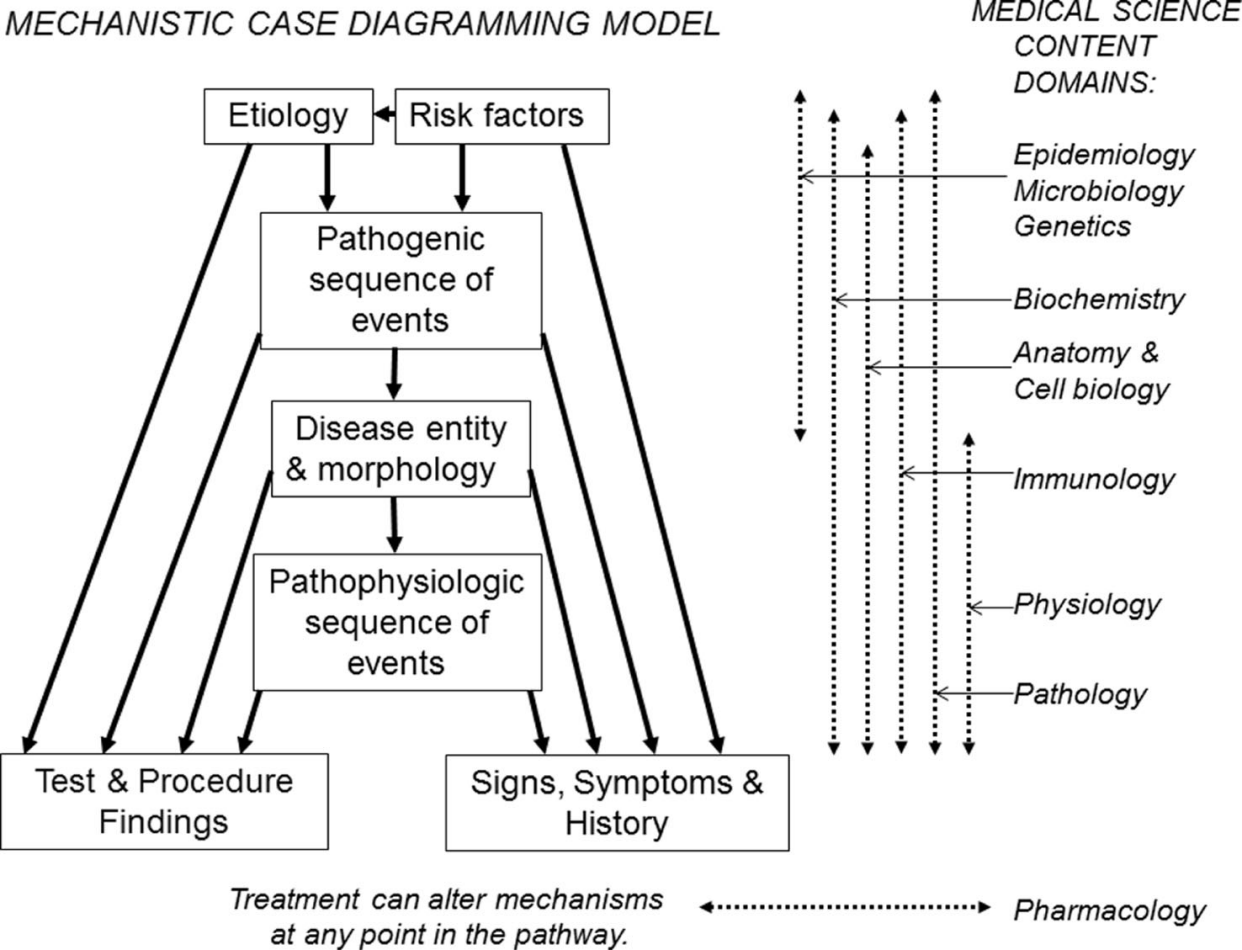
The hierarchical structure of a mechanistic case diagram starts with the etiology and risk factors, which then lead to the disease entity through several pathogenic sequences of events. The disease then leads to clinical findings, including signs and symptoms, and abnormal radiologic and laboratory findings, via several pathophysiologic sequences of events. Basic medical science content domains that correlate with the various mechanisms in the model are illustrated at the right.

A model for ‘mechanistic case diagramming’ is illustrated in Fig. 1. A major characteristic of mechanistic case diagraming (MCD) is that risk factors, disease entity and morphology, signs and symptoms, and test and procedure findings used in creating the mechanistic flow diagram are derived from those found in a specific case scenario. The diagramming exercise is then completed by filling in the etiologic, pathogenic, and pathophysiological sequence of events that explain the findings in the case. This process of connecting pathogenic and pathophysiological mechanisms with findings in a case, that students have just analyzed and discussed, brings heightened clinical relevance to the associated medical science (basic science) concepts.

Mechanistic case diagrams differ from classical concept maps, which are ‘graphical tools for organizing and representing knowledge’ in reference to a ‘focus question’ or subject area (4). A recent review of concept maps clarified that mechanistic case diagrams differ from concept maps in that they ‘do not include the action words between nodes (keywords and phrases) that are essential components of concept mapping; nevertheless, they (mechanistic case diagrams) promote understanding of pathobiological processes, and the basis for a patient's clinical presentation’ (5). Hence, mechanistic case diagrams are more focused than concept maps in that their arrow connections have a limited and unidirectional mechanistic meaning, that is, causes, leads to, results in, develops into, predisposes to, is followed by, and so on. It is this lack of complexity that allows mechanistic case diagrams to be efficiently created by students and scored by instructors as they teach important pathogenic and pathophysiologic mechanisms. Little or no training is needed for instructors and new students to understand the expectations of MCD.

This report will describe our enhancements to the original pedagogic design of mechanistic case diagrams as proposed by Engelberg and Guerrero, and then describe the 6-year experience at Iowa in their implementation in several teaching environments, including small group learning, independent learning exercises, and team-based learning (TBL). Our enhancements and modifications include web-based automation of diagram creation and scoring, pre-determination of items to be diagramed, feedback by comparison of student diagrams with an instructor's, and objective scoring. Several of these were previously piloted in a variant modification of MCD in collaboration with Kumar et al. at the University of New South Wales (6). The additional modifications at Iowa have resulted in accessible and efficient construction of pathogenic and pathophysiological mechanistic case diagrams by students (~15 min/diagram), ease of presentation and discussion of diagrams in a small group or lecture, efficiency of diagram exercise creation by faculty, and high satisfaction ratings by students and faculty.

## Methods

**Fig. 2:**
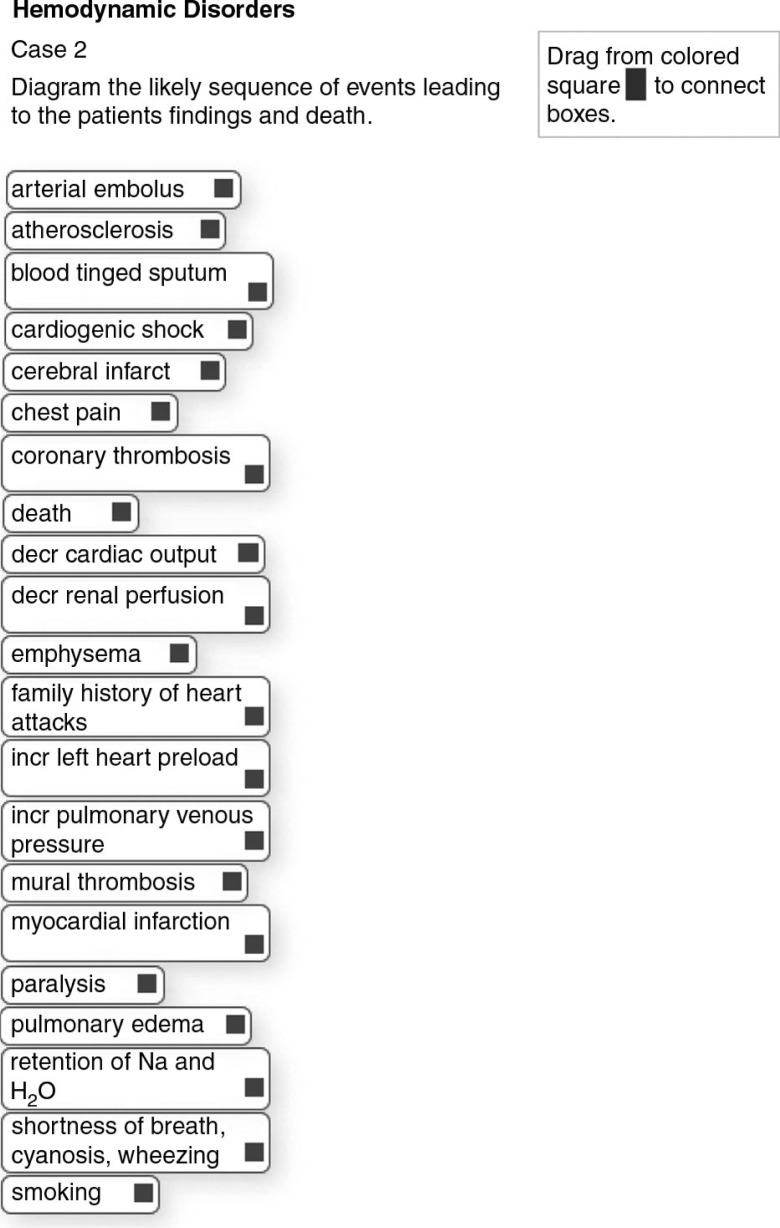
This is the opening screen for the case in Table 1 before diagramming starts.

**Fig. 3:**
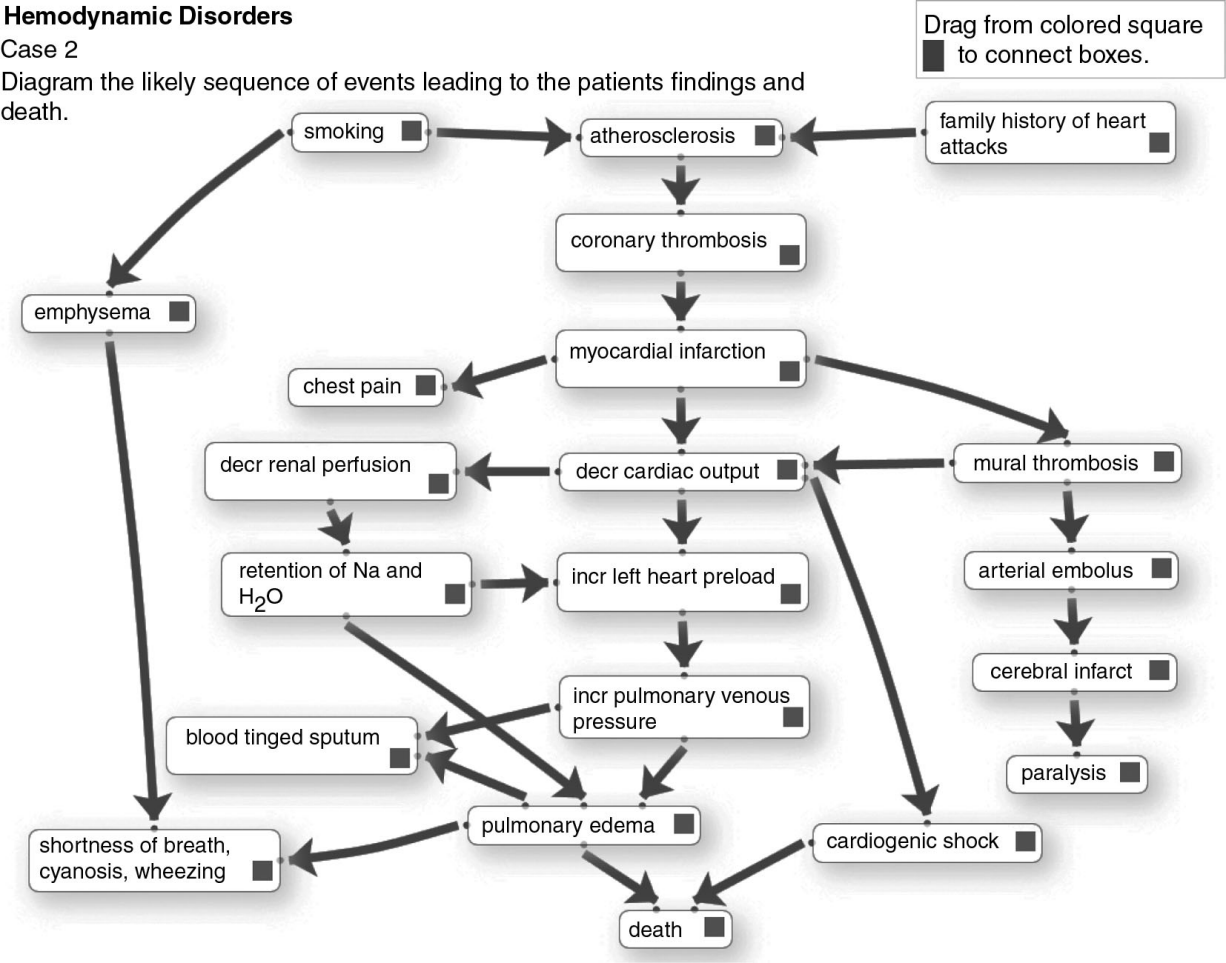
This is the ‘ideal’ completed diagram.

An example of the development of a mechanistic case diagram using the web-based electronic format is illustrated in Table 1, and Figs. 2 and 3. To create an exercise, the instructor first chooses an appropriate case scenario, which can be one already in use in the curriculum (Table 1). The case scenario will generally consist of a history, including symptoms and risk factors, physical findings, and additional findings such as test and procedure results, radiological images, and gross and histopathologic images of biopsy and/or autopsy specimens, plus follow-up findings or outcomes if appropriate. Next the instructor creates an item list that is composed of the risk factors and clinical signs and symptoms, test and procedure findings, and in this case also autopsy findings, that appear in the case scenario. The instructor then adds to the list those additional items that they want the students to learn about and use in making their diagram. These will be items in the pathogenic sequence of events, items for associated disease entity(ies), and items in the pathophysiologic sequence of events. To be manageable, the list should contain approximately 15–22 items and definitely no more than 25 items. The instructor then adds all the items to the web-based case editor. Figure 2 shows the opening screen of the student interface with the alphabetized list of items generated for the case from Table 1.

**Table 1:** Example of a case scenario (abbreviated here) used in the hemodynamic disorders unit

History: This 66-year-old man was admitted with a 24-hour history of chest pain and acute shortness of breath and blood tinged sputum. He was a heavy smoker and had a family history of heart disease.
Physical findings: Vital signs: pulse 140/min, respirations 42/min, BP 80/50 mm Hg. He was cyanotic with moist crackles and wheezes over both lung fields.
Laboratory findings: Chest x-ray (which is consistent with pulmonary edema)
Clinical course: In spite of appropriate treatment, 2 days later he suddenly became aphasic and paralyzed on the right side of his body. Renal output progressively decreased and he died 2 days later.
Autopsy findings: Gross image of heart (which shows atherosclerosis, coronary thrombus, acute myocardial infarct and a mural thrombus) Gross image of lung (which shows marked pulmonary edema) Microscopic of lung (which shows pulmonary edema and mild emphysema) Examination of the brain was not permitted.

### Student interface and diagram creation

The student will initially study the clinical, laboratory, radiological and pathologic findings in the case scenario and answer any associated study questions. Then, to create a diagram the student accesses the interface shown in Fig. 2 via the intranet with their unique ID, arranges items on the stage in a logical order, and connects them with arrows. To show a reciprocal effect, arrows can be placed in opposite directions between items. Arrows can cross over each other. There may be multiple arrows leading to and from items. Items can be moved around on the stage and the arrow connections will follow the items. Arrows can be deleted and re-drawn. As items are moved to the stage and arrows are drawn, their coordinates are written to a database, from which the diagram can be re-generated. This allows the student to save a partially completed diagram and return later to finish it. Then in class they can access, display and discuss their completed diagram via the web interface. A completed student diagram for the case in Fig. 2 might resemble the diagram in Fig. 3. Note that as per the model in Fig. 1, etiology and risk factors are positioned at the top; the main disease processes (e.g., myocardial infarct) near the middle; and the signs and symptoms, and test results at the end of the pathways.

### Instructor's ‘solution’ diagram

After the instructor enters a case list as shown in Fig. 2, they then create an instructor ‘solution’ diagram, which can then be saved to the database similar to the process described above. This solution diagram can be linked to a web-based facilitator's manual to assist group leaders. It can also be used as student feedback after the student finalizes their diagram. Finally, it can be used for automated scoring of students’ diagrams by electronically calculating the percent of student arrow connections that match the Instructor's.

**Fig. 4:**
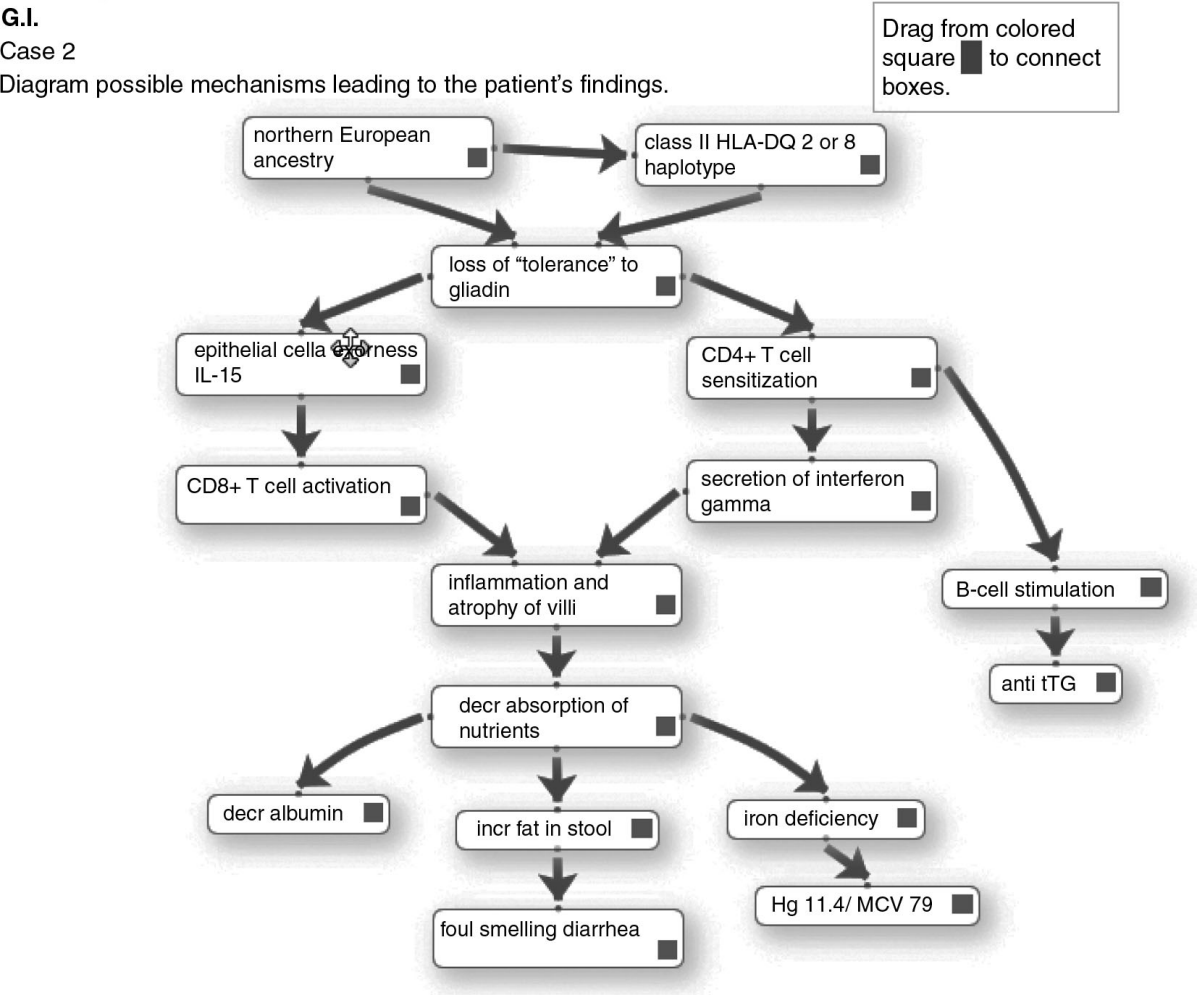
Case-based mechanistic diagram of a patient with celiac disease. Note the emphasis in this case on immunopathogenic mechanisms.

Instructors can create diagrams with a high level of complexity in that there may be numerous arrows per item or arrows depicting feedback mechanisms. By their choice of items on the list, instructors can choose to place greater emphasis on the pathophysiology of clinical findings, as in the diagram in Fig. 3 or on pathogenesis of the disease entity as in a completed diagram from another case shown in Fig. 4. Finally, instructors may add distractors to the list that do not belong in the pathway for the disease, so that students then have to decide to leave them unused at the margin.

### Technology

The diagramming interface illustrated in Figs. 2–4 will work with most popular browsers and includes the iPad and many mobile devices. The application is scripted using PHP on the server and requires JavaScript be enabled on the client computer. The JavaScript portion of the application uses the libraries JQuery and JQuery_ui. The jsPlumb library provides a way to visually connect the elements displayed. The touch adaptation for tablet devices is provided by jQuery UI Touch Punch. Upon submission of the diagram, the user name, the diagram name and the positions of the diagram boxes and their arrow connections are recorded on the server in a MySQL database. See Acknowledgements for further details.

The above describes the technology of our second-generation diagramming application, which is currently in use in our curriculum. The first-generation application, which was used for the first 3 years of implementation, was created by an SWF file using action script within Adobe Flash, this was the application with which we calculated some of the quality control evaluation data described later in the paper. The graphical user interface for automated scoring in the second-generation application described earlier is also functional.

## Implementation and evaluation at Iowa

Computerized MCD exercises were first implemented in Iowa in 2009 in small groups of second-year students in the medical pathology curriculum for (~156 students, 8 students per group). Extending over two semesters, there are currently 63 case scenarios for small group presentations and discussions. These include history and physical, and radiological, laboratory, and pathologic materials. Students study the cases ahead of class and prepare to present and discuss them in small group sessions. For 44 of these 63 cases, they also have to prepare ahead of class an associated mechanistic case diagram that they will present after all of the case findings are discussed in class. The diagram is intended to provide a review discussion of etiology, risk factors, disease entities, and intervening pathogenic and pathophysiologic mechanisms that have led to the patient's clinical and pathologic findings.

After an initial pilot implementation in two groups of eight students in 2009, the 16 students were asked to anonymously rate how well the diagramming exercises facilitated their learning of pathogenic and pathophysiologic mechanisms and how efficiently the diagrams could be created. See evaluation scores in Table 2. All 16 responding students strongly agreed or agreed that the diagramming exercises facilitate understanding of pathogenic and pathophysiologic mechanisms, and none disagreed. All but two students strongly agreed or agreed that time and effort spent in creating the diagram before class was efficient or very efficient. Two students felt the time spent was acceptable and none felt the time spent was excessive. Reported median time to create a diagram for a case was 15 min with a range of 10–45 min.

**Table 2:** Evaluation scores

Group responding	Evaluation stem	Score (scale: 4=excellent or strongly agree; 3=good or agree)
*n*=16/16 students (in pilot)	Effectiveness for learning of pathogenic and pathophysiologic mechanisms … Efficiency of time and effort spent in creating the diagram before class …	3.6/4.0 3.4/4.0
*n*=134/156 students (after full implementation)	Effectiveness for learning … Efficiency of time spent learning …	3.5/4.0 3.2/4.0
*n*=11/20 facilitators of small group (after full implementation)	Effectiveness and efficiency in enhancing student learning …	3.4/4.0

Student's comments about the diagramming application in the pilot were uniformly positive. Representative comments follow: ‘… made describing pathophysiology clearer and more efficient … really helps guide our thinking … speeds up and makes pathogenesis much clearer … time well spent and very instructive … putting the diagram together is simple … it is figuring out the actual steps that takes time … it was fun’. The only suggestion for improvement was that two students wanted the option to add their own items to the list beyond the ones provided. Following full implementation of the 44 diagramming exercises in 20 small groups of eight students each, student evaluation scores remained very positive (see Table 2), and comments were very similar to the pilot.

Facilitator evaluations of the effectiveness and efficiency of use of the diagraming exercises were also in the good to excellent range (see Table 2). Recurrent positive comments from facilitators were predominantly positive: ‘Diagrams make the students think more about the pathophysiology of the case and how the clinical, laboratory and pathologic findings all fit together … gave students a place to start to try and figure out the terminology and helps them know what is important to present … a really great tool to give a visual framework for thinking through cases … helps me see how they think, and has uncovered some glaring misunderstandings that I think are important to discuss for the student's success in the course and beyond … they promote a more thorough discussion of the case … keeps them (and me) from wandering off into non-essential or erroneous details … helps them sort out the causality of events in disease … students like the opportunity to put the presenting symptoms together with the pathophysiology … diagrams have helped the student organize their thinking when approaching the cases … at the end of the case it serves to reinforce important concepts and address any lingering questions … a good sum up of the case after the student presentations; it reminds them of many of the important points of the case’. Constructive facilitator criticisms were: ‘We don't have sufficient time to do (the diagrams) and discuss the case … students are using them to cover all the “required” details rather than to help organize their thinking … useful in the first semester, but in the second the students need to be doing much more independent thinking’.

Student assessment in small group is by facilitator's subjective grading of a student's overall performance. Diagrams are not specifically scored for purposes of a grade. However, we were able to evaluate how well the application performed overall since all the student arrow connections were recorded in a database. In the first semester of the 2012 course, in order to do continuous quality improvement of our ongoing implementation of the diagramming exercises in the curriculum, we looked retrospectively at the performance of the class of 156 students on 30 diagrams, with five to six diagrams being created per student. The mean student performance was 65% correct (range 0–100%) compared to the arrow connections on the instructor ‘solution’ diagram, demonstrating that the cases were indeed challenging. We also estimated discrimination and reliability as we do routinely for examinations. Mean discrimination for the 30 exercises was 0.29 (range −0.14–+0.63). As part of a validity investigation, we found that correlation with their multiple choice (MCQ) class exam and with facilitators overall evaluation of student performance in a small group was 0.29 and 0.30, respectively. Both were statistically significant (*p*<0.05). This suggests that the skill involved in constructing a case diagram is related to, but not identical to that assessed with the MCQ exam. The ‘true’ score correlation was approximately 0.54. Reliability for the diagramming exercise calculated as a coefficient α across standardized case means was α=0.47 (corresponding to a G coefficient of 0.47 for five cases).

The above calculations are from a large number of students each taking a sample of cases as a low stakes ungraded assignment. Performance under more standard and higher stake conditions would likely generate higher reliabilities. Nevertheless, these results do suggest the possibility for use in a more controlled environment for objectively grading students. Further study is needed to better understand its potential in higher stakes assessment.

No formal controlled evaluation of increased learning from diagramming exercises has been done at Iowa; however, a preliminary study was carried out with a variant of the application described in this paper in collaboration with the University of New South Wales, which did demonstrate an increase in learning (6).

### Pilot implementation in independent learning and TBL

Based on the positive implementation within small groups in the pathology curriculum, we created diagramming exercises for use in 12 existing case studies in the endocrine physiology section of the human organ systems course, and in the biochemistry course. These 12 cases are used in these courses for independent study and self-assessment, and are a non-required supplement to lectures. The new diagramming exercises were incorporated into the case study so that after the student creates and finalizes their diagram, they are allowed to see the completed instructor diagram for comparison, but only after they achieve minimal percent correct connections. Since the cases are supplemental, only about 20% of ~150 students have used them each year, and student performance is highly variable. Nevertheless, satisfaction questionnaires from students who responded were similarly positive to small group satisfaction (4.5 on a 5 point scale).

We recently piloted the use of diagramming exercises in TBL sessions in biochemistry, again with promising results. Briefly, in the TBL mode, students are given an online case scenario, study questions, and a diagramming exercise. Before class, students individually study the case scenario and study questions, and complete but do not finalize the diagramming exercise. Then in the large classroom (after a short quiz and discussion of the case and study questions), student groups are asked to work together to choose the best student diagram in their group, making some modifications if they wish. After 10 min, the instructor calls on groups at random to send their group's presenter to the podium to demonstrate to the whole class their diagram and the rationale for the arrow connections they have made.

## Discussion

Mechanistic cause and effect diagrams, not directly connected to case scenarios, have been utilized in medical education since the early 1970s, where they have been used to illustrate a wide variety of disease processes. For example, Robbins’ *Pathologic Basis of Disease*, 1st edition, Figure 3.28, is one of the earliest mechanistic diagrams to appear in textbooks (7). Such diagrams now abound in pathology and pathophysiology textbooks; pathology textbooks focusing on pathogenesis of disease entities and pathophysiology textbooks focusing more heavily on pathways that explain clinical findings. As noted in the introduction, Engelberg in 1992, and Guerrero in 2001, took mechanistic cause and effect diagrams a step further and made them case specific (1, 2).

In pathology, small group case discussion sessions at Iowa from 1992 to 2002, we experimented with the use of hand drawn mechanistic case diagrams on the chalkboard, to facilitate discussion of the how and why (pathogenesis) of the disease represented in the case under discussion, and of the pathophysiology underlying the clinical findings resulting from the disease. We also introduced short case-based mechanistic diagramming questions in progress exams. Based on end-of-course satisfaction questionnaires, students felt that the diagramming exercises in examinations significantly modified their study patterns, stimulating them to not only acquire factual information but also understand cause and effect associations (presented at the AAMC Annual Meeting, New Orleans, October 1998). However, these items are very time consuming to grade and there was perceived lack of objectivity in grading, so they were phased out. However, still believing that mechanistic case diagrams play an important role in facilitating teaching of pathogenic and pathophysiologic mechanisms, in 2009 it became feasible to develop the technology for computerizing and implementing the diagramming exercises, as described above in methods.

In the original descriptions of MCD, Guerrero and Azar had students develop the item lists themselves for diagramming cases for PBL sessions (2, 3). However, we decided to have students work from instructor generated item lists. Some might justifiably argue that an instructor created list provides an element of clueing that can prevent open-ended thinking on the part of the student. On the contrary, providing the student with a list of pathogenic and pathophysiologic events has the advantage of constraining the focus to those concepts the instructor feels are most important to convey and at what depth of granularity. The instructor-generated list can also direct the discussion to the review of specific and clinically meaningful integration of basic concepts that students have been exposed to in concurrent lectures or in other courses. Because students may come up with very different items and levels of granularity that may or may not be within the scope of the course, the instructor generated lists define the level of granularity appropriate for discussion. Equally important is that a pre-set list of items to diagram markedly increases the speed and efficiency of diagramming. By constraining the task, we have found that students can study up to four case scenarios a week and create accompanying diagrams. This makes it possible to cover a broad range of disease processes in a short period of time. For example in our current course students are required to work up and discuss 63 cases over the course of two semesters, with 44 of these having associated diagramming exercises. Finally, we believe pre-set lists to diagram can increase the reliability of the pathway diagramming exercises by precisely defining the task for students, thus assuring that all are responding to the same perceived task. This will potentially make objective scoring feasible if future research confirms a high level of consensus/agreement between expert-generated diagrams created for the same case. Further, as shown in the study at UNSW, providing lists as well as pre-populating the stage with some items and arrows would even further speed up the creation of diagrams as well as the ability to reliably score results (6).

The advantages of web-based delivery of diagramming exercises are similar to those achieved by moving any pedagogy to electronic delivery. For diagramming exercises, these include efficiency of editing by faculty, efficiency and accessibility for students, the ability for students to save and re-access their work for display in classroom, and the potential to do automated feedback and scoring.

### Other possible venues

We envision that mechanistic case diagrams could have a role in returning to the basic sciences in the clinical years as self-paced independent learning exercises. This is in keeping with the LCME standard of measuring basic science grounding in the clinical years. However, for this to be achieved with MCD exercises, rigorous outcomes research needs to be carried out. We also envision that mechanistic diagrams could be used to facilitate discussion of pharmacological and other known interventions that can interrupt the mechanisms depicted in the diagrams to produce an altered outcome. Also, in graduate student level pathogenesis of disease courses, mechanistic case diagrams could potentially be used as a clinically relevant way to discuss gaps in our knowledge of causal relationships and interventions, and hypotheses for investigating these gaps to increase scientific knowledge.
